# PanACEA: a bioinformatics tool for the exploration and visualization of bacterial pan-chromosomes

**DOI:** 10.1186/s12859-018-2250-y

**Published:** 2018-06-27

**Authors:** Thomas H. Clarke, Lauren M. Brinkac, Jason M. Inman, Granger Sutton, Derrick E. Fouts

**Affiliations:** 1 J. Craig Venter Institute, Rockville, MD 20850 USA; 2 Department of Biotechnology and Food Technology, Durban University of Technology, Durban, 4000 South Africa

**Keywords:** Pan-genome, Pan-chromosome, Visualization, Viewer, PanOCT, fGR, fGI

## Abstract

**Background:**

Bacterial pan-genomes, comprised of conserved and variable genes across multiple sequenced bacterial genomes, allow for identification of genomic regions that are phylogenetically discriminating or functionally important. Pan-genomes consist of large amounts of data, which can restrict researchers ability to locate and analyze these regions. Multiple software packages are available to visualize pan-genomes, but currently their ability to address these concerns are limited by using only pre-computed data sets, prioritizing core over variable gene clusters, or by not accounting for pan-chromosome positioning in the viewer.

**Results:**

We introduce PanACEA (Pan-genome Atlas with Chromosome Explorer and Analyzer), which utilizes locally-computed interactive web-pages to view ordered pan-genome data. It consists of multi-tiered, hierarchical display pages that extend from pan-chromosomes to both core and variable regions to single genes. Regions and genes are functionally annotated to allow for rapid searching and visual identification of regions of interest with the option that user-supplied genomic phylogenies and metadata can be incorporated. PanACEA’s memory and time requirements are within the capacities of standard laptops. The capability of PanACEA as a research tool is demonstrated by highlighting a variable region important in differentiating strains of *Enterobacter hormaechei.*

**Conclusions:**

PanACEA can rapidly translate the results of pan-chromosome programs into an intuitive and interactive visual representation. It will empower researchers to visually explore and identify regions of the pan-chromosome that are most biologically interesting, and to obtain publication quality images of these regions.

**Electronic supplementary material:**

The online version of this article (10.1186/s12859-018-2250-y) contains supplementary material, which is available to authorized users.

## Background

Next-generation sequencing technologies and a realization that single reference genomes are insufficient to grasp species-level diversity have resulted in a phenomenal rise in the number of publicly available bacterial genome sequences. A comparison of just six strains of *Streptococcus agalactiae* demonstrated that many more isolates are needed to capture strain diversity and helped define the concept of the bacterial pan-genome: the set of genes (core and variable) that are encoded within a bacterial species [1]. Tools have been developed to perform multiple genome comparisons by computing orthologous gene clusters and the resulting sets of core and variable genes [2–10]. Chan et al. extended the pan-genome concept to the “pan-chromosome”, where the order and orientation of core genes produce a consensus circular scaffold; thus, providing the framework for placing variable genes into discrete “flexible genomic regions (fGRs)” [11]. It is these fGRs that help define phenotypic subspecies differences [12] and provide the means for survival under iron limiting conditions, host immune pressure, and antibiotics [11].

To facilitate the interpretation of results for biological discovery, visualization tools have been developed, but still suffer from a number of caveats. A subset of pan-genome visualization tools are web-based (which is good for human intuitive data representation, but poses costly overhead), but only work with pre-computed and/or static data and do not allow user-supplied sequence data [13–17]. Pan-Tetris [18] and PanViz [19] are both interactive, but do not easily display variable (a.k.a., flexible) genomic islands (fGIs) [11]. Some visualization tools focus on alignments of core regions [20], require complicated database dependencies or produce complicated network diagrams [21]. None of the existing pan-genome visualization tools are geared toward a standalone (i.e., client side), intuitive, pan-chromosome-based interactive browser that will enable researchers to navigate to those parts of the pan-genome that are most relevant to understanding strain-specific differences that may impact pathogenesis, antimicrobial resistance, and general fitness in a given environment.

Here we introduce PanACEA (Pan-genome Atlas with Chromosome Explorer and Analyzer), an open source standalone computer program written in PERL that generates locally-computed (client side) JavaScript-driven interactive web-pages to view pan-chromosome data generated by PanOCT [4] or other pan-genome clustering tools. It consists of multi-tiered views with circular representations of chromosome(s)/plasmid(s) containing selectable and user-configurable colored functional gene annotations/ontologies and zoomed-in linear illustrations of per genome fGI content in the fGRs located throughout the pan-chromosomes. The program can also produce views of multiple-sequence alignments of user-specified clusters and phylogenetic trees that can be colored based on the presence/absence of user-specified regions. Lastly, PanACEA can export publication-quality (SVG) or draft-quality image (PNG) images of any view, text tables, and the nucleotide or protein sequences of cluster members or representatives. This software was developed with the goal of being an intuitive, easy-to-use, standalone viewer that will empower researchers with the ability to visualize those regions of the pan-chromosome of their choosing that are of most biological interest. The identification of these regions and their surroundings will advance the understanding of the biology of these organisms and how they evolve by proving a much needed tool to comprehend those genomic differences that lead to increased antibiotic resistance, pathogen outbreaks, and differences in patient outcomes.

## Implementation

PanACEA is written in PERL and utilizes the BioPerl module to read in phylogenies. The PanACEA PERL scripts output HTML, JSON, and JavaScript files that are viewable with multiple web browsers, including Google Chrome (v 63.0), Mozilla Firefox (v 58.0.1), Apple Safari (v 11.0.3), and Internet Explorer/Edge (v 11.0.9600.18816/38.14393.1066.0). The scripts also use the MSAViewer [22] to display multiple sequence alignments. All resulting output files and functionalities, except for the MSAViewer, can be used offline.

## Results

### Data input

**Fig. 1:**
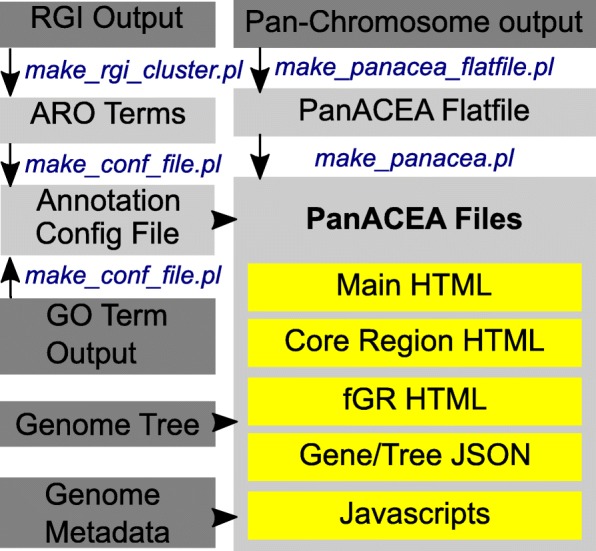
PanACEA Pipeline Flowchart. The PanACEA pipeline with the initial files shown in dark gray, the PanACEA PERL scripts shown in blue font, the resulting PanACEA intermediate files shown in light gray, and the final files shown in yellow. The final PanACEA output includes all the HTML pages, JSON files, and Javascripts scripts necessary to run the viewer. The RGI output referenced is generated by the RGI software package. Additional information on the requirements for the input files can be found in the user manual located on the GitHub page

PanACEA uses PERL scripts and a tab-delimited human-readable flat file that contains the following necessary information for the script to generate platform-independent visualizations: the gene order of the pan-chromosome “assemblies”, including the flexible and core regions (such as output of gene_order.pl [11]); detailed information about each gene; and the location of the sequences of the genes. Though this file can be recreated ad hoc and the user manual does provide descriptions, the PanACEA software package includes a script designed to translate the output of pan-genome software packages to the PanACEA flat file (Fig. 1). Currently, PanACEA must be downloaded or cloned from the GitHub site and run locally. As such, the flat file input provides flexibility for the user independent of which pan-genome generation software they wish to use, both current and future programs. Currently, PanACEA optimally works with PanOCT [4] and gene_order.pl [11] output (both are availible at https://sourceforge.net/projects/panoct/). An example dataset consisting of the PanOCT and gene_order.pl derived pangenome of 19 *Acinetobacter baumannii* genomes along with GO term and ARO term based gene annotations is also available at the PanACEA GitHub repository.

Beyond generic input requirements, PanACEA is highly configurable, allowing for customization of input features specific to the needs and available data of the researcher. Additional information, such as that describing the functionality of the genes or the relationship between genomes, can be incorporated (Fig. 1). Any functional annotation (i.e., Gene Ontology (GO) [23, 24] or Antibiotic Resistance Ontology (ARO) [25] terms) can be added modularly through a configuration file that will associate colors with functional annotation as well as ontology information. Included with the package are scripts that will add annotation to the gene clusters in a format that PanACEA can read. For sets of genomes with a known evolutionary relationship, a Newick-formatted phylogenetic tree file can also be added, along with metadata information about the genomes such as isolation date, host, serotype, pathogen/non-pathogen, etc.

### Visualization features

The PanACEA interface enables the interactive exploration of pan-genomic data through multiple spatial views, from broad pan-chromosome/scaffold context through multi-gene regions to single gene details (Additional file 1: Figure S1). Pan-scaffold representations can be cyclic or linear and highlight flexible and core regions, with core genes individually colored by protein function. For cyclic representations, the nucleotide position coordinate system of the consensus pan-chromosome is used. The pan-scaffolds are shown at identical heights, independent of the number of genomes found in each region. For ease of differentiating short flexible and core regions, the flexible regions are all shown at staggered instances of three-quarters height, again regardless of how many genomes are contained in that region. Regions of interest, such as those involved in antibiotic resistance, virulence, bacteriophage, plasmid, or any other user-configured high-level feature can be preferentially displayed. Likewise, the pan-scaffold (main) page contains a table listing regions, genes, and specific functional terms and can be selected to also highlight the location of the genes. The main page includes a text search function to facilitate identifying specific genes and regions in the table and a zoom function on the top of the main page. The user can scale from the pan-scaffold to a more detailed view of single regions, whether a set of core genes or a fGR, either by clicking on the region on the pan-scaffold map or in the table. On separate pages, PanACEA provides a linear representation of gene context, associated functional annotation, and prevalence of the region in each genome. Given the possible complexity of a fGR, the display can be trimmed to focus on a reduced set of fGIs of interest. Additionally, when included, the genomic phylogeny, accessible from the fGR and core region pages, as well as the gene pages, enables phylogenomic analysis of any region of interest overlaid with user-provided metadata. This functionality can be extended to individual gene summary pages, which display gene annotation and provide access to sequence data and single gene analysis tools such as multiple sequence alignments. All PanACEA displays can be exported as publication-quality SVGs or preview graphics files in other formats (e.g., PNG) and the gene and region lists in tabular data as text files.

A more detailed description of both the PanACEA software package and the web pages with the visualization, complete with examples and help pages, is available in the PanACEA manual on the GitHub site.

### Use case

**Fig. 2:**
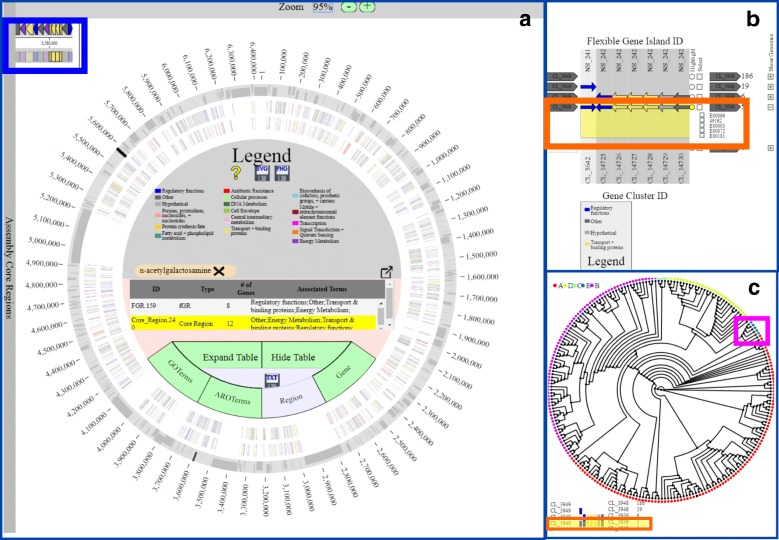
PanACEA Views of *E. hormaechei gat* and *aga* Operons. The PanACEA pan-chromosome images (**a**), fGR view (**b**), and phylogeny (**c**) showing the *gat* operon that can differentiate *E. hormaechei* subsp*. hormaechei* from other subsp. [12]*.* The location of the fGI in **b** and **c** is highlighted with the orange box*.* The default coloring scheme is shown in (**a**) with variable regions in dark gray and core regions in light gray. The variable regions are also shown at 0.75 height and on alternating sides of the chromosome to help differentiate small neighboring regions. The bounding core region that contains the *aga* operon is shown in the preview panel highlighted by the light blue box in **a**. The cluster of genomes containing the *gat* operon fGI are annotated as E and are highlighted in the genome phylogeny in **c** using the pink box. The images in **b** and **c** are derived from PNGs downloaded directly from the website. Additional information about the visualization can be found in the user manual located on the GitHub page

The biological utility and output of PanACEA is illustrated using the *Enterobacter hormaechei* pan-genome data generated from PanOCT from 219 genomes where PanACEA helped to visualize fGIs responsible for the known metabolic differences historically used to classify *E. hormaechei* subspecies [12]. The time to generate all necessary files from the PanOCT output to the final web pages was 466 s. In addition to the pan-genome, annotation files for each of the gene clusters calculated using GO terms and anti-microbial resistance genes from the CARD database using RGI were used [24, 25]. All the *E. hormaechei* PanACEA files are available on the GitHub site. The fGR depicted contains two GIs (one flexible and one core between core gene clusters 3936 and 3949) and encodes metabolic pathways historically used to define phenotypic differences between *E. hormaechei* subspecies (Fig. 2). *E. hormaechei* subsp. *hormaechei* is distinguishable from *E. hormaechei* subsp. *oharae* and *E. hormaechei* subsp. *steigerwaltii* by growth on dulcitol (a.k.a. galactitol) as the sole carbon source via the *gat* operon [26]. In contrast, *E. hormaechei* subsp. *oharae* and subsp. *steigerwaltii* both encode a different fGI (the *aga* operon) for the metabolism of N-acetylgalactosamine [27] (Fig. 2). We readily identified and located the genes and regions of interest by inputting “N-acetylgalactosamine” in the text search and selecting the highlighted regions and genes of interest in the main pan-chromosome view as shown in Fig. 2, thus allowing for analysis of the positional context. The output demonstrates the capability of PanACEA to highlight differences between strains in a visually informative manner and present the users with publication-ready images.

## Discussion

The memory and time usage required by the PanACEA scripts to run does not exceed the capabilities of most laptops, as shown in Additional file 1: Table S1. We compared runs of pan-chromosomes generated from between 20 and 219 genomes. The compute times ranged from 80 to 456 s, while the memory usage varied from 208 Mb to 3.16 Gb. We further found that increasing the number of fGR paths also lead to an increase in these requirements - surprisingly somewhat independent of number of genomes. For instance, the 193 *E. coli* genome pan-chromosome has almost twice as many fGR paths compared to a 219 *E. hormaechei* genome pan-chromosome and showed relative increases in time and memory usage. However, this increase is limited to a few minutes in terms of the CPU and a few gigabytes in terms of memory usage.

The modularity of PanACEA also allows for more functionality to be added. Further possible functions that can be included in future versions of PanACEA may include: multiple region views where genomes can be compared across neighboring fG and Core regions; additional gene annotation on the core region images, such as three letter gene names; graphs and text demonstrating the prevalence of different gene order and gene prevalence in clusters of genomes with the available metadata; and finally, to write additional scripts to transform the output from other pan-genome tools such as Roary [6] so that it can be used as input for PanACEA.

## Conclusions

PanACEA is an interactive visualization tool that leverages bacterial genomic data for the analysis of pan-genomes in the context of a consensus pan-chromosome. Its browser interface displays customizable annotation features such as the anti-microbial resistance and gene ontologies, which expedite the point-and-click exploration of pan-chromosomes when compared to text files and previous visualizations that lacked contextual browsing of variable regions. Its hierarchical design enables the navigation of both detailed and high level views of the data. The search and zoom functions permit users to identify genes and regions of interest and view these regions in the context of the full pan-chromosome, zoomed in close, or in the detail views in another window, as shown in our use case. PanACEA is database independent and browser agnostic, easy to install, and works off generalized flat files promoting interoperability across pan-genome software.

## Availability and requirements

**Project name**: PanACEA.

**Project home page:** https://github.com/JCVenterInstitute/PanACEA/

**Operating system(s)**: Platform independent.

**Programming language**: PERL, HTML, Javascript.

**Other requirements:** PERL v5.22.1, BioPerl v1.007001.

**License:** GNU GPL.

**Any restrictions to use by non-academics:** none.

## Additional file

Additinal file 1: **Table S1.** Memory and CPU time requirement of multiple PanACEA runs on a 2.3GHz Linux VM. **Figure S1.** PanACEA HTML page flowchart. (DOCX 22 kb)

## Abbreviations

ARO: Antibiotic Resistance Ontology
fG: flexible genomic
fGI: flexible genomic island
fGR: flexible genome region
GI: Genomic Island
GO: Gene Ontology
RGI: Resistance Gene Identifier
