# Supplementary material for: PanACEA: a bioinformatics tool for the exploration and visualization of bacterial pan-chromosomes
Source: BMC Bioinformatics. 2018 Jun 27;19:246. doi: 10.1186/s12859-018-2250-y (PMC6020400; doi:10.1186/s12859-018-2250-y)
Supplement: Supplementary file 1 — Table S1. Memory and CPU time requirement of multiple PanACEA runs on a 2.3GHz Linux VM. Figure S1. PanACEA HTML page flowchart. (DOCX 22 kb) [file 12859_2018_2250_MOESM1_ESM.docx]

**Additional file 1**

**Table S1. Memory and CPU time requirement of multiple PanACEA runs on a 2.3GHz Linux VM**

| **Script** | **Wall-clock Time*** | **CPU Time*** | **Peak Memory Usage*** |
| --- | --- | --- | --- |
| *make_panacea_flatfile.pl* | 2.534s/37.116s/27.310s | 1.545s/36.733s/26.276s | 206.539M/2.753G/2.775G |
| *make_rgi_clusters.pl* | 0.600s/0.498s/0.560s | 0.071s/0.084s/0.080s | NA - negligible |
| *make_conf_file.pl* | 0.451s/0.166s/0.458s | 0.084s/0.082s/0.083s | NA - negligible |
| *make_panacea.pl* | 75s/419s/322s | 8.072s/82.871s/33.498s | 279.629M/3.159G/351.172M |

**results are shown for pan-genomes created from 20/193/219 genomes*

**Figure S1. PanACEA HTML Page Flowchart.** Graphic showing the different views available on the PanACEA viewer, with the different levels of views (Chromosome, Region and Gene levels) indicated by the background color. All PanACEA images are screen shots generated from the PanOCT results from *Enterobacter hormaechei* as described in [1]. Transitions that result in new HTML pages/tabs are indicated by gray arrows, and transitions that merely alternate views on the same page/tab are indicated by green arrows. On the Chromosome page, the user can alternate between the chromosome view (i) and the plasmid view (ii). The chromosome view also has the ability to highlight regions and genes in the image based on the functions listed the legend (e.g. “Antibiotic Residence” as shown in iii) and on terms listed in the table (e.g. “DNA Transcription” as shown in iv). Likewise, previews of the region pages can be seen by mousing over the image, or clicking on the gene or region listed in the table (demonstrated in light blue box in Figure 2A). The region pages, including the Core Regions (v) and the Flexible Genomic Regions (vi), are accessible from the Chromosome page. The Core Region page contains a phylogeny where the presence of the core genes in different genomes can be interrogated. For the fGR page, a separated page with the phylogeny (vii) is rendered. From all the region, chromosome, and plasmid pages (i-vi), the html pages describing a single gene cluster are accessible. The single gene cluster pages contain several views, including a table containing: a summary of the gene cluster (including functional annotation, length, and centroid sequence) (viii); a multi-sequence alignment based on MSAViewer (ix) [2]; and a phylogeny showing the presence of the gene in the genomes (x). More detailed information on the use of PanACEA output can be found in the manual available at: https://github.com/JCVenterInstitute/PanACEA.

**References**

1. Chavda KD, Chen L, Fouts DE, Sutton G, Brinkac L, Jenkins SG, et al. Comprehensive Genome Analysis of Carbapenemase-Producing *Enterobacter* spp.: New Insights into Phylogeny, Population Structure, and Resistance Mechanisms. mBio. 2016;7:e02093-16.

2. Yachdav G, Wilzbach S, Rauscher B, Sheridan R, Sillitoe I, Procter J, et al. MSAViewer: interactive JavaScript visualization of multiple sequence alignments. Bioinformatics. 2016;32:3501–3.
